# WEE1 Inhibition in Combination With Targeted Agents and Standard Chemotherapy in Preclinical Models of Pancreatic Ductal Adenocarcinoma

**DOI:** 10.3389/fonc.2021.642328

**Published:** 2021-03-25

**Authors:** Sarah J. Hartman, Stacey M. Bagby, Betelehem W. Yacob, Dennis M. Simmons, Morgan MacBeth, Christopher H. Lieu, S. Lindsey Davis, Alexis D. Leal, John J. Tentler, Jennifer R. Diamond, S. Gail Eckhardt, Wells A. Messersmith, Todd M. Pitts

**Affiliations:** ^1^ Division of Medical Oncology, Department of Medicine, University of Colorado Anschutz Medical Campus, Aurora, CO, United States; ^2^ Department of Oncology, Dell Medical School, The University of Texas Austin, Austin, TX, United States

**Keywords:** pancreatic ductal adenocarcinoma, WEE1, DNA damage, 5-Fluorouracil (5-FU), irinotecan

## Abstract

Pancreatic ductal adenocarcinoma (PDAC) is a highly lethal cancer with high incidences of p53 mutations. AZD1775 (adavosertib, previously MK-1775) is a small molecule WEE1 inhibitor that abrogates the G2M checkpoint and can potentially synergize with DNA damaging therapies commonly used in PDAC treatment. The purpose of this study was to identify combination partners for AZD1775, including standard chemotherapy or targeted agents, in PDAC preclinical models. Low powered preliminary screens demonstrated that two of the four PDX models responded better to the combinations of AZD1775 with irinotecan or capecitabine than to either single agent. Following the screens, two full powered PDAC PDX models of differing p53 status were tested with the combinations of AZD1775 and irinotecan or capecitabine. The combinations of AZD1775 and SN38 or 5-FU were also tested on PDAC cell lines. Cellular proliferation was measured using an IncuCyte Live Cell Imager and apoptosis was measured using a Caspase-Glo 3/7 assay. Flow cytometry was conducted to measure alterations in cell cycle distribution. Western blot analysis was used to determine the effects of the drug combinations on downstream effectors. In PDX models with mutated p53 status, there was significant tumor growth inhibition from the combination of AZD1775 with irinotecan or capecitabine (*P* ≤ 0.03), while PDX models with wild type p53 did not show anti-tumor synergy from the same combinations (*P* ≥ 0.08). The combination of AZD1775 with SN38 or 5-FU significantly decreased proliferation in all PDAC cell lines, and enhanced apoptosis in multiple cell lines. Cell cycle distribution was disrupted from the combination of AZD1775 with SN38 or 5-FU which was recorded as G2M arrest and decreased G1 phase. AZD1775 inhibited phospho-CDC2 and increased the expression of **γ**H2AX that was either maintained or enhanced after combination with SN38 or 5-FU. The combination of AZD1775 with irinotecan/SN38 or capecitabine/5-FU showed anti-tumor effects *in vivo* and *in vitro* in PDAC models. These results support further investigation for these combination strategies to enhance outcomes for PDAC patients.

## Introduction

Pancreatic ductal adenocarcinoma (PDAC) is the fourth leading cause of cancer death in men and women, with approximately 35% of patients presenting with locally advanced disease at diagnosis (1). Despite advances in surgical techniques, radiation, and chemotherapy, the 5-year survival rate is one of the lowest at just 10% (2). Current therapeutic options for advanced disease include fluorouracil, leucovorin, irinotecan, and oxaliplatin (FOLFIRINOX) or gemcitabine plus nab-paclitaxel, however these therapies are limited by a generally short duration of response and cumulative toxicities (3, 4). Activation of the DNA damage response (DDR) pathway in cancer cells with chemotherapy can lead to treatment resistance (5). Targeting the cell cycle checkpoint has potential to decrease the activation of the DDR and to sensitize PDAC cells to chemotherapy or targeted agents for improved patient outcomes.

PDAC tumors frequently have mutated p53 which commonly results in a defective G1 checkpoint, thus forcing cancer cells to rely primarily on the G2M checkpoint to repair DNA damage before mitosis (6–8). Previous studies have reported that the highly selective, small molecule WEE1 inhibitor AZD1775 (adavosertib, previously MK-1775) can abrogate the G2M checkpoint, thereby forcing damaged DNA through mitosis (6, 9). WEE1 kinase regulates the G2M checkpoint by phosphorylating CDC2 in response to DNA damage (6, 10). Inhibition of WEE1 prevents the arrest of damaged DNA, which enhances CDC2 activity and drives cells in S phase to prematurely enter mitosis before repair (10). AZD1775 has also been shown to enhance sensitization to chemotherapy and antimetabolites in cancer cells with wild type p53, which indicates the beneficial effects of this compound are not dependent on dysfunctional p53 (11). Phase 1 clinical data has shown AZD1775 is clinically viable and can safely be combined with chemotherapies in advanced solid tumors (12).

It is plausible that AZD1775 may synergize with other DNA damaging agents utilized for PDAC treatment. The topoisomerase inhibitor irinotecan is a standard of care agent that has been shown to be effective for the treatment of several cancers including PDAC (13, 14). The combination of irinotecan with AZD1775 has shown synergy when tested in colorectal cancer cell lines and in other solid refractory tumors (15, 16). Capecitabine is an oral pyrimidine antimetabolite that passes intact through the intestinal wall and is locally converted to 5-fluorouracil (5-FU) in tumor tissue where the cytotoxic effects are activated (17). Previous studies have demonstrated capecitabine is effective for the treatment of pancreatic cancer, and the combination of AZD1775 with capecitabine has shown synergy in other tumor types (18, 19). Targeting WEE1 in combination with DNA damaging agent offers a promising therapeutic option for PDAC.

The purpose of this study was to use an unbiased *in vivo* screening and validation approach to identify efficacious combination partners for AZD1775 in preclinical models of PDAC using active chemotherapy agents and targeted drugs available through the National Cancer Institute’s Cancer Therapy Evaluation Program (CTEP). The results from this study suggest that AZD1775 may have clinical applications in PDAC when combined with irinotecan or capecitabine.

## Methods

### PDAC Patient-Derived Xenografts

All animal work was performed with approval by the University of Colorado Anschutz Medical Campus IACUC. Patient-derived tumor samples were collected from consenting PDAC patients at the University of Colorado Cancer Center with approval by the Colorado Multiple Institutional Review Board. These samples were used to generate patient-derived xenograft (PDX) models as described previously (20). Female athymic nude mice (aged 4-8 weeks) were purchased from Envigo (Indianapolis, IN) and implanted subcutaneously on the hind flanks with tumors sized approximately 3 mm^3^. Mice were randomized into treatment groups and treatments were initiated when the average tumor volume reached between 100-300 mm^3^. In the initial PDAC drug screen, mice (*n* = 3/group) were treated with AZD1775, navitoclax, irinotecan, romidepsin, olaparib, AZD8186, gemcitabine, and the combination of these agents with AZD1775. Percent tumor growth inhibition (TGI) values were calculated using the end of study mean tumor volumes (MTV) of the vehicle and treatment groups with following equation: (1 – (MTV_vehicle_/MTV_treated_) × 100). Drugs with TGI values greater in the combination than both single agents in at least two PDX models were selected for further *in vivo* validation. The mutational profiles of PDX models were assessed using the ArcherDX FusionPlex Solid Tumor panel, and mutations are indicated in the figure legend’s superscript.

Two PDAC PDX models (*n* = 10 mice/group) were expanded per drug combination. Treatments included AZD1775 (50 mg/kg, PO, QD, in 0.5% hydroxypropylcellulose), irinotecan (15 mg/kg, IP, QW), capecitabine (60 mg/kg, PO, twice weekly, in corn oil), navitoclax (100 mg/kg, PO, thrice weekly, in 10% ethanol, 30% PEG400, 60% Phosal 50PG), and the combination of AZD1775 with these agents. Mice were monitored daily for signs of toxicity, and tumor volume and weight were measured twice weekly using digital calipers and a scale. Tumor volumes were calculated using the following equation: volume = (length × width^2^) × 0.52.

### Cell Lines and Reagents

PDAC cell lines BxPc-3, MiaPaca-2, and Panc1 were purchased from ATCC and routinely screened for mycoplasma. The PDAC cell line L3.3 was a generous gift from John’s Hopkins University. All cells were cultured in DMEM (Corning) supplemented with 10% FBS (Atlas Biologicals), 1% penicillin-streptomycin, and 1% MEM nonessential amino acids (Corning). All PDAC cells tested were p53 mutants. Cells were maintained at 37˚C in an atmosphere containing 5% CO_2_. AZD1775 was provided by AstraZeneca or purchased from MolPool (Hong Kong) depending on availability during the study. Irinotecan was purchased from the University of Colorado Hospital Pharmacy. The active metabolite of irinotecan, SN38, was purchased from Sigma for *in vitro* analyses. Capecitabine and navitoclax were purchased from Active Biochem. The active metabolite of capecitabine, 5-fluoruracil (5-FU), was purchased from the University of Colorado Hospital Pharmacy for *in vitro* analyses.

### Cell Proliferation and Apoptosis

The anti-proliferative and apoptotic effects of AZD1775, SN38, and 5-FU were evaluated on PDAC cell lines. AZD1775 concentrations were selected based on a proliferative CellTiter-Glo assay (Promega) and previously reported data by others (21). Cells were seeded at optimal density in 96-well white walled plates, allowed to adhere for 24 hours, and then treated with AZD1775 (125 nM and 250 nM), SN38 (10 nM), 5-FU (2.5 µM), and the combination of AZD1775 with these agents. Proliferation was measured every 2-4 hours for 72 hours in an IncuCyte ZOOM live cell imager (Essen Biosciences). Percent confluence was analyzed using the IncuCyte ZOOM 2018A software. Synergy was calculated from the 72 hour proliferation averages using the Bliss Additivity model as previously described (22). Apoptosis was assessed using the Caspase Glo 3/7 assay (Promega) as per manufactures instructions. For apoptosis, PDAC cells were seeded in 96-well white walled plates and treated for 24 and 48 hours as described above with AZD1775, SN38, and 5-FU. Luminescence was measured using a Synergy H1 microplate reader (Biotek) for CellTiter-Glo and Caspase 3/7.

### Cell Cycle Analysis

Alterations in cell cycle distribution were measured with flow cytometry. PDAC cell lines were seeded at optimal density in 6-well plates and treated with AZD1775, SN38, and 5-FU as described above for 24 and 48 hours. The cells were then washed in PBS, suspended in Krishan’s stain, and incubated at 4°C for 24 hours. Cells were analyzed for cell cycle and ploidy using flow cytometry by the University of Colorado Cancer Center Flow Cytometry Core Facility.

### Immunoblotting

The effects of AZD1775, SN38, and 5-FU on downstream effectors were measured by immunoblotting. PDAC cell lines were seeded at optimal density in 6-well plates and treated with AZD1775, SN38, and 5-FU as described above. After 24 and 48 hours of drug exposure, cells were rinsed with PBS, lysed with ice cold RIPA buffer containing protease and phosphatase inhibitors, and scraped on ice. The cell lysates were collected, sonicated, and centrifuged at 4˚C, 12,000 × g for 10 minutes. Protein concentrations were measured using the Pierce BCA Protein Assay kit (Thermo Fisher, Rockford, IL). Protein lysates were boiled in Laemmli sample buffer, run on 4% to 12% Bis-Tris precast gels (Thermo Fisher, Rockford, IL), and transferred to nitrocellulose membranes using the Pierce G2 FastBlotter (Thermo Fisher, Rockford, IL). The membranes were blocked for 1 hour at room temperature and probed with primary antibodies with rocking at 4˚C overnight. Primary antibodies CDC2, phospho-CDC2, γH2AX, phospho-histone H3, and α-tubulin were purchased from Cell Signaling Technology (Danvers, MA) and were diluted per company instructions. After incubating overnight, the membranes were washed three times for 5 minutes with TBS/Tween20 before probing with anti-rabbit and/or mouse DyLight IgG secondary antibodies purchased from Cell Signaling Technology (Danvers, MA, diluted 1:15,000). The blots were imaged using the Odyssey Infrared Imaging System (Licor, Lincoln, NE).

### Statistical Analysis

Results from the proliferation, apoptosis, and cell cycle experiments were analyzed for statistical significance using GraphPad Prism 8.1 (San Diego, CA) using an unpaired t-test. *In vivo* models were analyzed with an unpaired t*-*test with Welch’s correction. Differences were determined to be statistically significant with *P*-values ≤ 0.05.

## Results

### *In Vivo* Effects of AZD1775 Combinations in PDAC PDX Models

**Figure 1:**
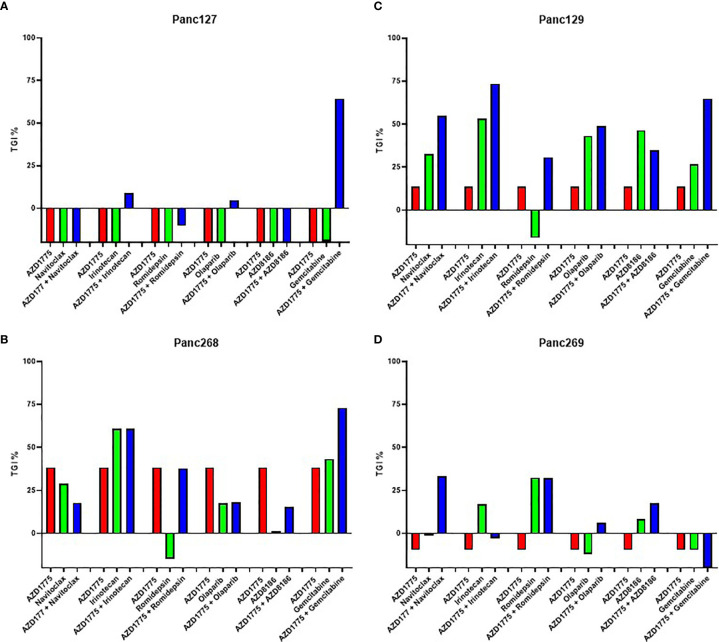
Effect of AZD1775 alone or in combination with chemotherapy or targeted agents in pancreatic ductal adenocarcinoma (PDAC) PDX models. Percent tumor growth inhibition (TGI) values were calculated for each model (*n* = 3 mice/group). AZD1775, 50 mg/kg (PO, QD); navitoclax, 100 mg/kg (PO, QWx3); irinotecan, 15 mg/kg (IP, QW); romidepsin, 1.34 mg/kg (IP, QW); olaparib, 50 mg/kg (PO, QD); AZD8186, 25 mg/kg (IP, QD); gemcitabine, 40 mg/kg (IP, QW). **(A)** Panc127, **(B)** Panc268 (p53^MT^, KRAS^MT^), **(C)** Panc129 (p53^MT^, KRAS ^MT^), **(D)** Panc269 (p53^MT^, KRAS^MT^, CDKN2A^MT^).

Four PDAC PDX models were utilized for the initial low-powered, unbiased *in vivo* screen to evaluate the efficacy of AZD1775 alone and in combination with navitoclax, irinotecan, romidepsin, olaparib, AZD8186, and gemcitabine. Gemcitabine enhanced the anti-tumor effects of AZD1775 in three of the four models as shown by increased TGI values in the combination. However, this combination was not selected for further validation by our group because of the previously published data by others and the number of ongoing clinical trials (1, 9, 23–25). In at least two of the four PDAC PDX models tested, the combination of AZD1775 with irinotecan or navitoclax increased the TGI values, therefore these drug combinations were selected for further validation in full powered p53 mutant (MT) and wild type (WT) PDX models (**Figure 1**). A similar screen from our lab group in triple negative breast cancer models reported increased TGI values from the combination of AZD1775 and capecitabine in two models, therefore this combination was also selected for further validation in full powered PDAC models (21).

**Figure 2:**
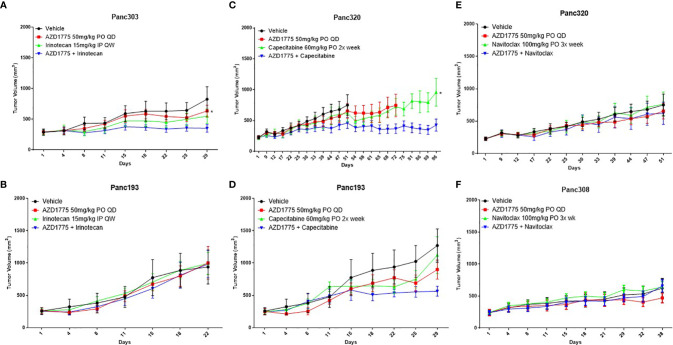
Effect of AZD1775 alone or in combination with irinotecan, capecitabine, or navitoclax in PDAC PDX models of varying p53 status (*n* = 10 mice/group). **(A)** Panc303 (p53^MT^, KRAS^MT^), **(B, D)** Panc193, **(C, E)** Panc320 (p53^MT^, KRAS^MT^, SMAD4^MT^, CDKN2A^MT^), **(F)** Panc308 (p53^MT^, KRAS^MT^, SMAD4^MT^). Statistical significance between the single agent and combination is indicated next to the single agent (* = p < 0.05).

In the subsequent PDX experiments, the combination of irinotecan and AZD1775 significantly inhibited tumor growth relative to single agent AZD1775 in Panc303 (p53^MT^, *P* = 0.02) (**Figure 2A**). No significant differences were observed in Panc193 (p53^WT^, *P* ≥ 0.96) from the combination of irinotecan and AZD1775 (**Figure 2B**). The combination of capecitabine and AZD1775 significantly inhibited tumor growth relative to capecitabine in Panc320 (p53^MT^, *P* = 0.03) and trended to decrease tumor growth in Panc193 (p53^WT^, *P* = 0.08) (**Figures 2C, D** **)**. Although the initial screen showed the combination of navitoclax and AZD1775 inhibited tumor activity, this combination did not result in a significant reduction of tumor growth in Panc320 or Panc308 (p53^MT^, *P* ≥ 0.15) (**Figures 2E, F** **)**. Additionally, the navitoclax combination was not well tolerated, and caused poor body condition and thrombocytopenia in mice which was observed as petechiae. Due to this adverse reaction, we did not pursue the combination of navitoclax and AZD1775 for further *in vitro* validation.

### Anti-Proliferative Effects of AZD1775 Combinations on PDAC Cell Lines

**Figure 3:**
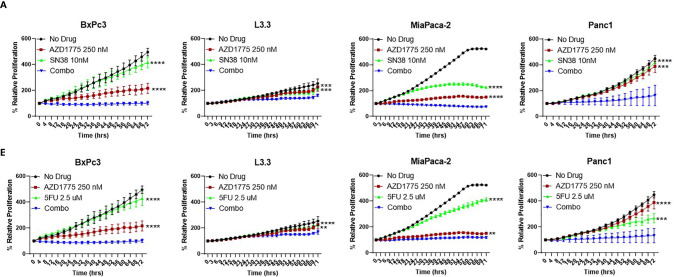
Anti-proliferative effects of AZD1775 (250 nM) and **(A)** SN38 or **(B)** 5-FU in PDAC cell lines. BxPc3 (p53^MT^), L3.3 (p53^MT^), MiaPaca-2 (p53^MT^), and Panc1 (p53^MT^) were treated with AZD1775 and SN38 or 5-FU, and proliferation was measured over a 72 hour period using the IncuCyte Zoom with data normalized to hour 0. Data were analyzed with a t-test to compare single agents to the combination (** = *p* ≤ 0.01, *** = *p* ≤ 0.001, **** = *p* ≤ 0.0001). Statistical significance between the single agent and combination is indicated next to the single agent.

To better understand the combination effects witnessed *in vivo*, we utilized several *in vitro* experiments to elucidate potential mechanisms of action. The anti-proliferative effects of AZD1775, SN38, and 5-FU alone and in combination with AZD1775 were assessed on PDAC cells using the IncuCyte Zoom over a 72 hour period. The combination of AZD1775 (250 nM) and SN38 or 5-FU significantly decreased (*P* ≤ 0.005) proliferation in all PDAC cell lines relative to either single agent (**Figures 3A, B** **)**. This trend was also observed with the combination of a lower dose of AZD1775 (125 nM), where the combination with SN38 or 5-FU significantly decreased (*P* ≤ 0.05) proliferation in all PDAC cell lines (**Supplemental Figure 1**). Bliss additivity calculations showed varying synergy from the combinations of AZD1775 at both concentrations with SN38 or 5-FU. The effects of AZD1775 with SN38 or 5-FU combinations demonstrated synergy in Panc1 and were moderately synergistic in BxPc3 and L3.3. The combination was additive in the MiaPaca-2 cell line. (**Supplemental Table 1**).

### Apoptotic Effects of AZD1775 Combinations on PDAC Cell Lines

**Figure 4:**
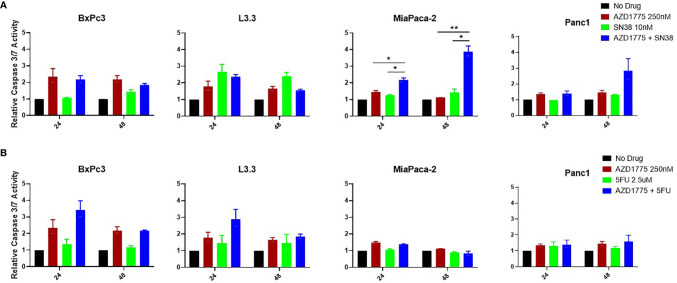
Apoptotic effects of AZD1775 (250 nM) and **(A)** SN38 or **(B)** 5-FU in PDAC cell lines. Cells were treated with AZD1775 and SN38 or 5-FU for 24 and 48 hours, and apoptosis was measured using a Caspase Glo 3/7 assay with data normalized to the No Drug control. Data were analyzed with a t-test to compare single agents to the combination (* = *p* ≤ 0.05, ** = *p* ≤ 0.01).

To determine the effects of AZD1775 on initiation of apoptosis, PDAC cells were exposed to AZD1775, SN38, and 5-FU at the previously described drug concentrations for 24 and 48 hours. Following drug exposure, the plated cells were analyzed for induction of apoptosis with a Caspase 3/7 Glo assay. The combination of AZD1775 (250 nM) with SN38 significantly increased (*P* ≤ 0.01) apoptosis in MiaPaca-2 at both time points measured (**Figure 4A**). There were no significant differences in apoptosis in BxPc3, L3.3, or Panc1 from the combination of SN38 and AZD1775 (250 nM) (**Figure 4A**). The combination of 5-FU and AZD1775 did not significantly impact apoptosis in any PDAC cell line after 48 hours (**Figure 4B**).

The combination of AZD1775 (125 nM) with SN38 increased apoptosis Panc1 after 24 hours and in MiaPaca-2 at both time points (*P* ≤ 0.03) (**Supplemental Figure 2A**). There were no significant differences in apoptosis in any PDAC cell line from the combination of AZD1775 (125 nM) with 5-FU (**Supplemental Figure 2B**).

### Effects of AZD1775 on Cell Cycle Arrest

**Figure 5:**
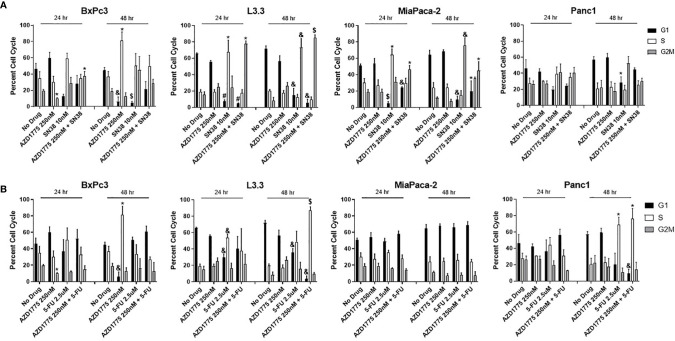
Cell cycle analysis of AZD1775 (250 nM) and **(A)** SN38 or **(B)** 5-FU in PDAC cell lines. Cells were treated with AZD1775 and SN38 or 5-FU for 24 and 48 hours, then cell cycle arrest was assessed using Krishan’s stain followed by flow cytometry. Data were analyzed with a t-test to compare single agents to the combination (* = *p* ≤ 0.05, & = *p* ≤ 0.01, $ = *p* ≤ 0.001, # = *p* ≤ 0.0001).

To determine if the combination of AZD1775 with conventional chemotherapy altered cell cycle dynamics, flow cytometry was performed on PDAC cell lines treated for 24 and 48 hours with AZD1775 (250 nM), SN38 (**Figure 5A**), and 5-FU (**Figure 5B**). The combinations of AZD1775 (125 nM) with SN38 (**Supplemental Figure 3A**) and 5-FU (**Supplemental Figure 3B**) were also analyzed. Single agent AZD1775 did not significantly alter cell cycle distribution in PDAC cells at either timepoint, with the exception of BxPc3. In BxPc3, AZD1775 (125 nM and 250 nM) decreased G2M phase (*P* < 0.02) after 24 hours, and after 48 hours decreased G1 phase (*P* < 0.003) and increased S phase (*P* < 0.04). The combinations of AZD1775 (125 nM and 250 nM) with SN38 decreased G1 phase (*P* < 0.02) and increased G2M phase (*P* < 0.03) relative to single agent AZD1775 in PDAC cell lines at both timepoints. These alterations in G1 phase distribution could have been driven by single agent SN38, which decreased G1 phase (*P* < 0.03) in L3.3 and MiaPaca-2 at both timepoints. Single agent SN38 also increased S phase (*P* < 0.03) arrest in L3.3 and MiaPaca-2 after 24 hours, though this increase was not recorded after 48 hours drug exposure in any of the PDAC cell lines. The combinations of AZD1775 (125 nM and 250 nM) with 5-FU decreased G1 phase (*P* < 0.003) and increased S phase (*P* < 0.02) arrest in L3.3 and Panc1 after 48 hours. The alterations in Panc1 cell cycle could be driven by the significantly increased S phase (*P* = 0.01) arrest from single agent 5-FU after 48 hours. Single agent 5-FU also decreased G1 phase (*P* < 0.004) at both timepoints and increased S phase (*P* = 0.002) after 24 hours in L3.3.

### Assessment of AZD1775 Targets and Downstream Effectors by Immunoblotting in PDAC Cell Lines

**Figure 6:**
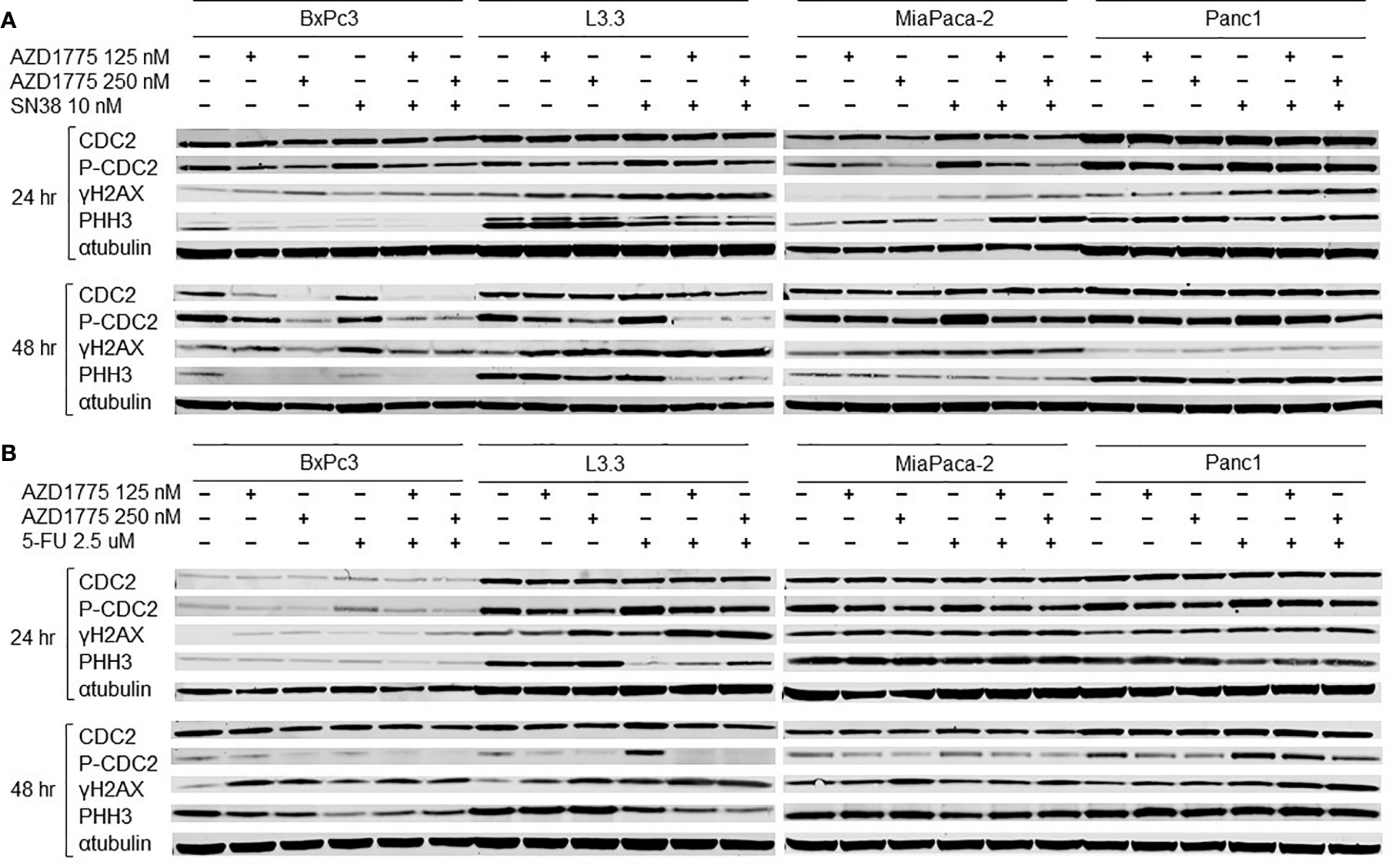
Effects of AZD1775 and **(A)** SN38 or **(B)** 5-FU on downstream effectors of DNA damage and cell cycle in PDAC cell lines. Cells were treated AZD1775 and SN38 or 5-FU for 24 and 48 hours then total protein was extracted. Expression of CDC2, p-CDC2, γH2AX, and PHH3 was measured with immunoblotting.

To determine the effects of AZD1775 and the other therapies on downstream effectors of inhibited WEE1, immunoblotting was performed after 24 and 48 hours of drug exposure. AZD1775 alone increased downstream effectors of DNA synthesis, while the combination of AZD1775 with either SN38 (**Figure 6A**) or 5-FU (**Figure 6B**) increased expression of DNA damage response markers. AZD1775 alone decreased expression of phospho-CDC2, a marker of mitotic delay, at both time points for BxPc3, MiaPaca-2, and Panc1, and after 48 hours in L3.3. While phospho-CDC2 expression increased after 24 and 48 hours exposure to SN38 or 5-FU, the addition of AZD1775 in combination treatments decreased phospho-CDC2. Additionally, γH2AX expression, a marker of DNA damage, increased in response to SN38 and the combination of SN38 and AZD1775 after 24 hours in L3.3, MiaPaca-2, and Panc1, and after 48 hours in all PDAC cell lines. This was not the case for 5-FU, where increases in γH2AX expression were driven by increasing doses of AZD1775. Overall, phospho-histone H3 expression, a marker of mitosis, decreased in PDAC cells after exposure to SN38 for 24 hours, though this was not observed after 48 hours exposure. Similarly, there was a decrease in phospho-histone H3 expression in response to 5-FU after 24 hours in L3.3 and Panc1, and after 48 hours in BxPc3, L3.3, and MiaPaca-2. The combinations of SN38 with AZD1775 and 5-FU with AZD1775 resulted in decreased phospho-histone H3 expression at both time points in L3.3.

## Discussion

Pancreatic ductal adenocarcinoma (PDAC) is one of the most lethal cancers in the United States, with a 5-year survival rate of only 10% (2). It has been suggested that cancer types with high occurrences of p53 mutations, such as PDAC where approximately 60% of patients have mutant p53, may experience increased sensitivity to WEE1 targeted drugs as a result of their functionally inactive G1/S checkpoints (https://www.cbioportal.org/). WEE1 is a key regulator of cell cycle progression at the G2M checkpoint and controls entry into mitosis. AZD1775, a potent WEE1 inhibitor, abrogates WEE1 by phosphorylating and inactivating CDC2 which can force cells to enter mitosis with unrepaired DNA damage (7). In this study, we first examined the combination of the WEE1 inhibitor AZD1775 with several targeted CTEP compounds and approved PDAC chemotherapies in a low powered *in vivo* screen. From the initial screening models, we observed AZD1775 synergized with irinotecan and navitoclax. A similar screen from our lab in triple negative breast cancer (TNBC) models showed synergy with the combination of AZD1775 and capecitabine (21). Considering the similar high incidence of mutant p53 in both PDAC and TNBC, we decided to test the combination of AZD1775 with irinotecan, capecitabine, and navitoclax in p53 mutant and wild type PDAC full powered cohorts. Following *in vivo* validation, navitoclax did not significantly alter tumor development and was not well tolerated, therefore only irinotecan and capecitabine analogues were tested for synergy with AZD1775 in PDAC cell lines.

Our results demonstrate that the combination of AZD1775 with irinotecan and capecitabine can enhance anti-tumor effects in PDAC PDX models and in PDAC cell lines. The combination of AZD1775 with SN38 or 5-FU significantly decreased proliferation in all PDAC cell lines and increased apoptosis in multiple cell lines. Lal et al. reported AZD1775 increased apoptosis and abrogated the G2M cell cycle checkpoint in PDAC cell lines (26). In the current study, the combination of AZD1775 with SN38 increased G2M arrest in PDAC cells, indicating accumulation of damaged DNA before forced entry into mitosis as a result of WEE1 inhibition treatment. The effect is especially notable in MiaPaca-2, where the combination of AZD1775 and SN38 increased G2M arrest and apoptosis at both measured timepoints, and increased phospho-histone H3 expression after 24 hours. Previous studies have demonstrated that an increase in phospho-histone H3 correlates with increased late stage in the apoptotic processes, and it suggests changes in chromosome condensation could have increased apoptosis in MiaPaca-2 (27, 28). Additionally, pre-clinical studies in colon cancer cells showed the combination of AZD1775 and 5-FU to decrease G1 and increase G2M phase (15). This is similar to what we observed in several of the PDAC models tested. Single agent AZD1775 inhibited the primary substrate of WEE1, phospho-CDC2, and increased expression of **γ**H2AX that was either maintained or enhanced after combination treatments, demonstrating activation of the DNA damage response pathway. These data are consistent with previous literature, where the combination of AZD1775 and 5-FU or gemcitabine caused similar alterations in phospho-CDC2 and **γ**H2AX expression in colorectal and pancreatic cancer cells (29, 30).

Although the trend of improved responses from the combination of a WEE1 inhibitor with DNA damaging treatments is apparent, the mechanism for this interaction is still unclear. Studies in colon and pancreatic cancer cell lines have reported that AZD1775 can decrease cell viability independent of combinations with DNA damaging agents (15, 16, 26). In contrast, in the present study PDAC cells were not significantly impacted by AZD1775 alone. It is possible that using a higher concentration of the WEE1 inhibitor could elicit a single agent response, although our results are supported by others who have reported single agent AZD1775 did not have an anti-proliferative effect even at a concentration of 300 nM (29). Recent publications have also debated the reliability of using p53 status as an indicator of PDAC sensitivity to these combinations. Hirai et al. demonstrated the combination of AZD1775 with DNA damaging antimetabolites and topoisomerase inhibitors could selectively potentiate antitumor activity in p53 mutant colorectal cancer cells (29). These results have been supported by research in several other cancer types, although other studies have reported both p53 mutant and p53 wild type cells respond to AZD1775 (10, 11, 23). In our *in vivo* cohorts, the combination of AZD1775 with irinotecan or capecitabine had anti-tumor effects on the p53 mutant models while no significant differences were noted for the p53 wild type models. These results are consistent with published reports of synergy in the combination of AZD1775 with capecitabine or irinotecan in solid tumors (18, 19, 31). Interestingly, there were no significant differences in tumor development from the combination of AZD1775 and navitoclax, though both PDX models tested were mutant p53. In diffuse large B-cell lymphoma, the combination of AZD1775 and navitoclax has been shown to induce cell death with up to a 10 fold decrease in cell viability recorded *in vitro*, however the authors acknowledged the limitations of this combination as the anti-apoptotic protein inhibitors have significant side effects and are poorly tolerated (32).

Clinical trials to evaluate the impact of AZD1775 alone or in combination with other standard of care therapies have shown promising results in PDAC and other cancer types. In a recent dose escalation study, PDAC patients treated with AZD1775 in combination with gemcitabine had a favorable overall survival result of 22 months compared to those receiving gemcitabine alone (24). In the study, Cuneo et al. also suggested the addition of AZD1775 may have enhanced the control over the primary tumor development. Patients in the study experienced a similar side effect profile to those from a trial by Do et al., where patients with solid refractory tumors had manageable hematologic and GI toxicities after treatment with single agent AZD1775 (33). AZD1775 has also been shown to be tolerable when combined with irinotecan in pediatric solid refractory tumors (31). In a recent phase II study, the addition of AZD1775 to carboplatin enhanced median progression free survival in patients with p53 mutant ovarian cancer (34). Consistently, biopsy and blood samples collected in these studies have reported decreased phospho-CDC2 activity and increased γH2AX activity after AZD1775 combination therapy.

Some researchers have suggested that inhibiting multiple targets along with WEE1 pathway could overcome the limitations observed from single agent AZD1775. The results from this experiment suggest the combination of AZD1775 with irinotecan and capecitabine may improve outcomes and could be promising combination partners for PDAC patients. Though p53 status may act as a predictive biomarker for certain treatment strategies, more reliable markers are necessary to treat future PDAC patients.

## Data Availability Statement

The original contributions presented in the study are included in the article/**Supplementary Material**. Further inquiries can be directed to the corresponding authors.

## Ethics Statement

The animal study was reviewed and approved by University of Colorado Anschutz Institutional Animal Care and Use Committee.

## Author Contributions

TP and SH conceived and designed the study. SB, BY, DS, and MM assisted with experiments. CL, LD, AL, JT, JD, GE, and WM provided tumors and/or technical contributions and advice in writing the manuscript. All authors contributed to the article and approved the submitted version.

## Funding

This work was supported by the National Institutes of Health (NIH) and the National Cancer Institute (NCI) through 5P30CA046934-25 (University of Colorado Cancer Center Support Grant), 1UM1CA186688 (JD, WM, and GE) and CPRIT Scholar Award #RR160093 (GE). The funding bodies played a role in study design, data collection, analysis, and interpretation.

## Conflict of Interest

The authors declare that the research was conducted in the absence of any commercial or financial relationships that could be construed as a potential conflict of interest.

## Supplementary Material

Supplementary Figure 1 Anti-proliferative effects of AZD1775 (125 nM) and **(A)** SN38 or **(B)** 5-FU in PDAC cell lines. PDAC cells were treated with AZD1775 and SN38 or 5-FU, and proliferation was measured over a 72 hour period using the IncuCyte Zoom with data normalized to hour 0. Data were analyzed with a t-test to compare single agents to the combination (* = *p* ≤ 0.05, ** = *p* ≤ 0.01, *** = *p* ≤ 0.001, **** = *p* ≤ 0.0001). Statistical significance between the single agent and combination is indicated next to the single agent.

Supplementary Figure 2 Apoptotic effects of AZD1775 (125 nM) and **(A)** SN38 or **(B)** 5-FU in PDAC cell lines. Cells were treated with AZD1775 and SN38 or 5-FU for 24 and 48 hours, and apoptosis was measured using a Caspase Glo 3/7 assay with data normalized to the No Drug control. Data were analyzed with a t-test to compare single agents to the combination (* = *p* ≤ 0.05, ** = *p* ≤ 0.01, *** = *p* ≤ 0.001).

Supplementary Figure 3 Cell cycle analysis of AZD1775 (125 nM) and **(A)** SN38 or **(B)** 5-FU in PDAC cell lines. Cells were treated with AZD1775 and SN38 or 5-FU for 24 and 48 hours, then cell cycle arrest was assessed using Krishan’s stain followed by flow cytometry. Data were analyzed with a t-test to compare single agents to the combination (* = *p* ≤ 0.05, & = *p* ≤ 0.01, $ = *p* ≤ 0.001, # = *p* ≤ 0.0001).

Supplementary Table 1 The synergistic effects of AZD1775 (125 nM, 250 nM) and SN38 or 5-FU in PDAC cell lines. Bliss additivity was calculated using the 72 hour proliferation data averages. Values greater than 1 indicate synergy between the two agents tested.
