# Supplementary figures and images for: WEE1 Inhibition in Combination With Targeted Agents and Standard Chemotherapy in Preclinical Models of Pancreatic Ductal Adenocarcinoma
Source: Front Oncol. 2021 Mar 25;11:642328. doi: 10.3389/fonc.2021.642328 (PMC8044903; doi:10.3389/fonc.2021.642328)

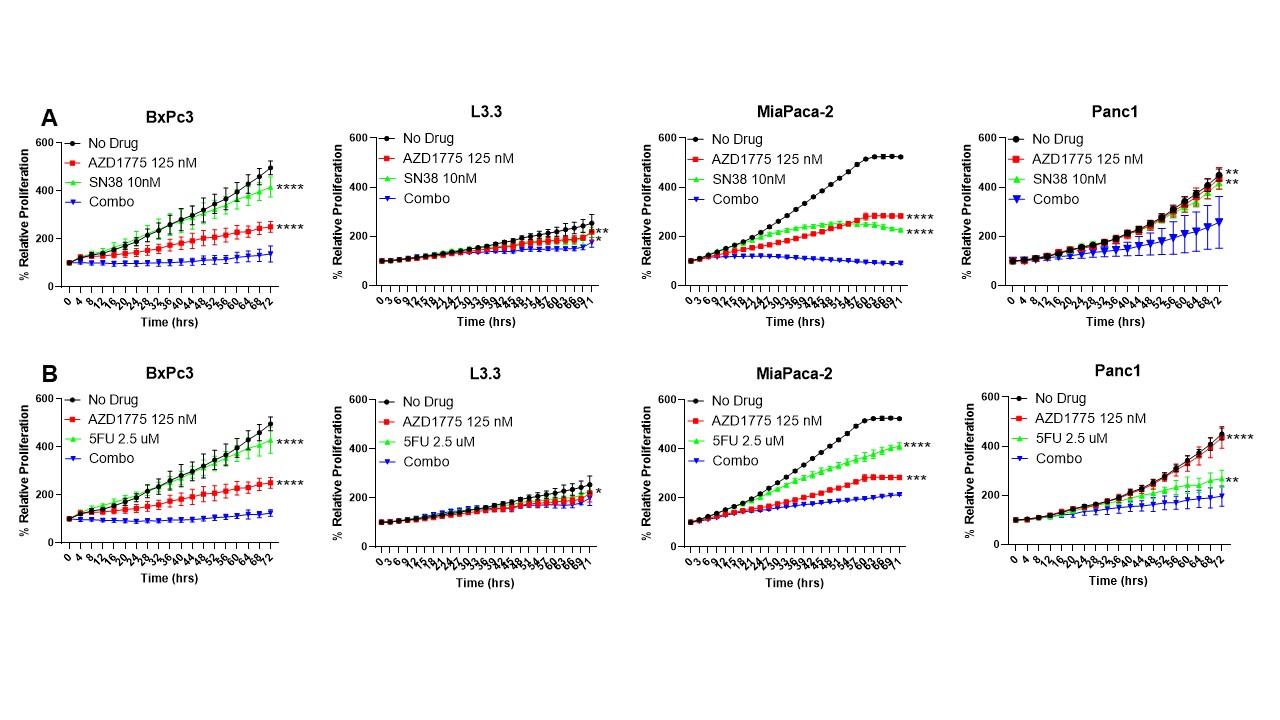

Supplement: Supplementary Figure 1 — Anti-proliferative effects of AZD1775 (125 nM) and (A) SN38 or (B) 5-FU in PDAC cell lines. PDAC cells were treated with AZD1775 and SN38 or 5-FU, and proliferation was measured over a 72 hour period using the IncuCyte Zoom with data normalized to hour 0. Data were analyzed with a t-test to compare single agents to the combination (* = p ≤ 0.05, ** = p ≤ 0.01, *** = p ≤ 0.001, **** = p ≤ 0.0001). Statistical significance between the single agent and combination is indicated next to the single agent. [file Image_1.jpg]

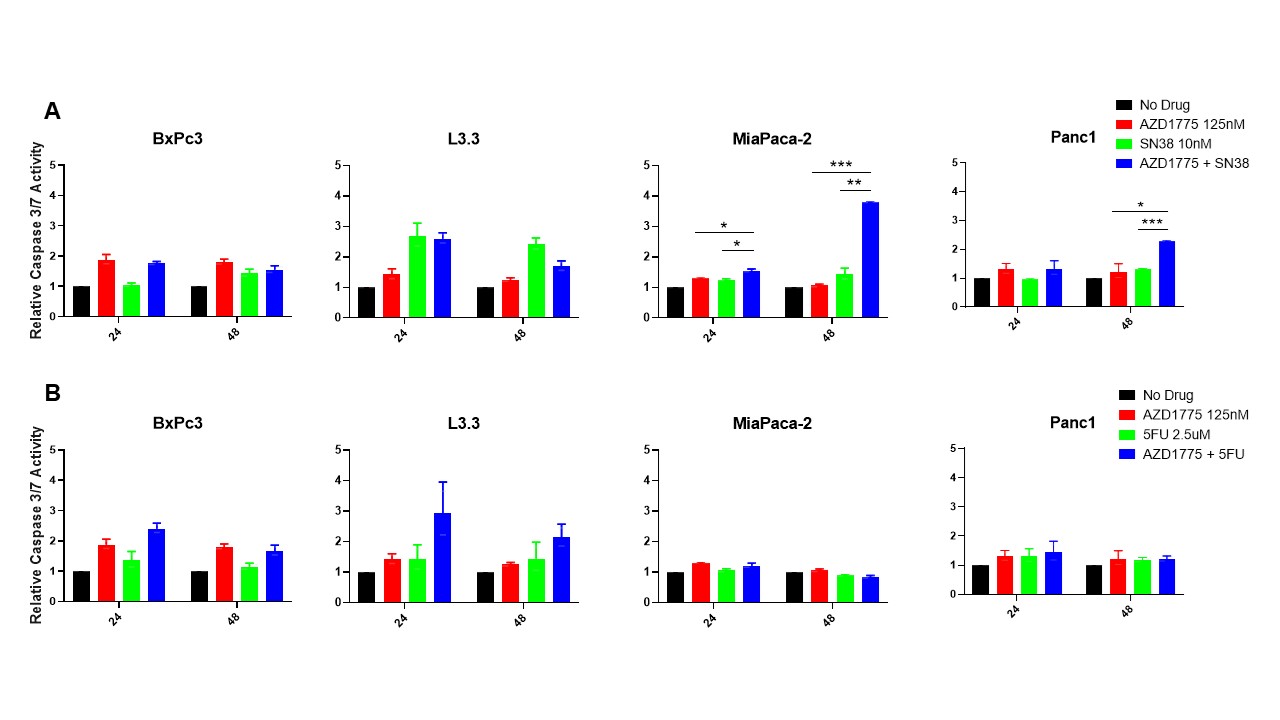

Supplement: Supplementary Figure 2 — Apoptotic effects of AZD1775 (125 nM) and (A) SN38 or (B) 5-FU in PDAC cell lines. Cells were treated with AZD1775 and SN38 or 5-FU for 24 and 48 hours, and apoptosis was measured using a Caspase Glo 3/7 assay with data normalized to the No Drug control. Data were analyzed with a t-test to compare single agents to the combination (* = p ≤ 0.05, ** = p ≤ 0.01, *** = p ≤ 0.001). [file Image_2.jpeg]

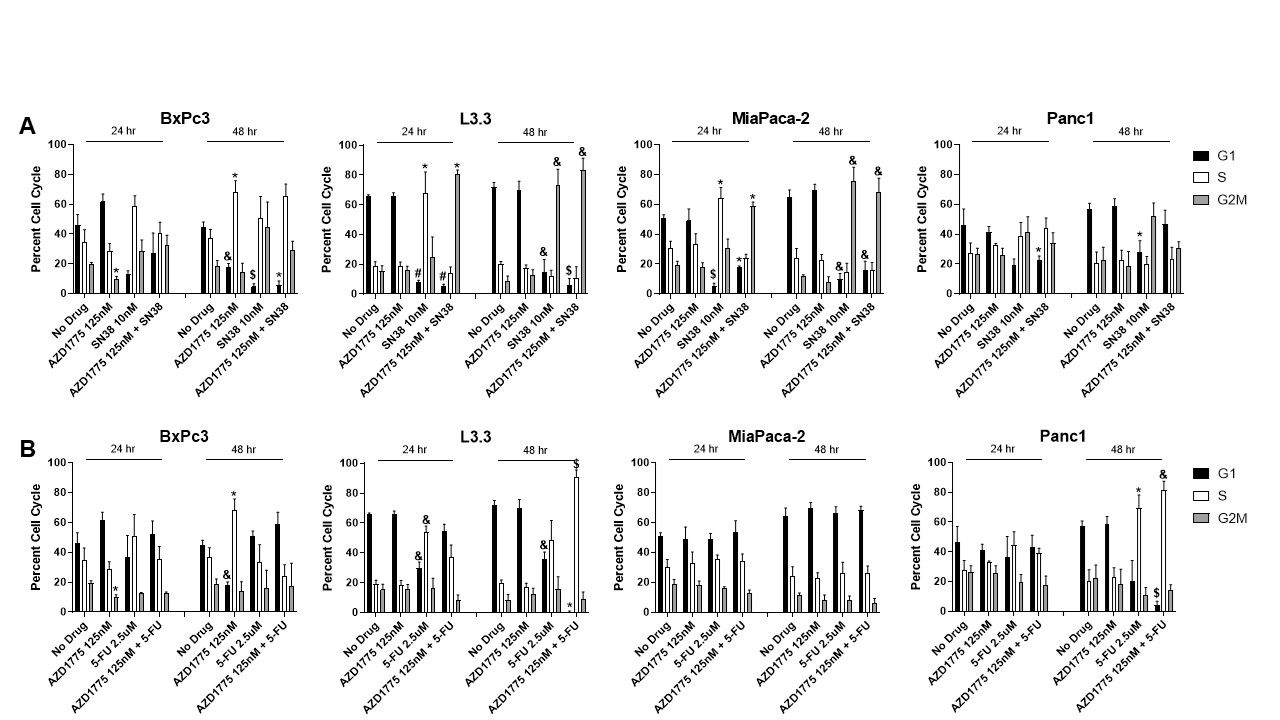

Supplement: Supplementary Figure 3 — Cell cycle analysis of AZD1775 (125 nM) and (A) SN38 or (B) 5-FU in PDAC cell lines. Cells were treated with AZD1775 and SN38 or 5-FU for 24 and 48 hours, then cell cycle arrest was assessed using Krishan’s stain followed by flow cytometry. Data were analyzed with a t-test to compare single agents to the combination (* = p ≤ 0.05, & = p ≤ 0.01, $ = p ≤ 0.001, # = p ≤ 0.0001). [file Image_3.jpeg]

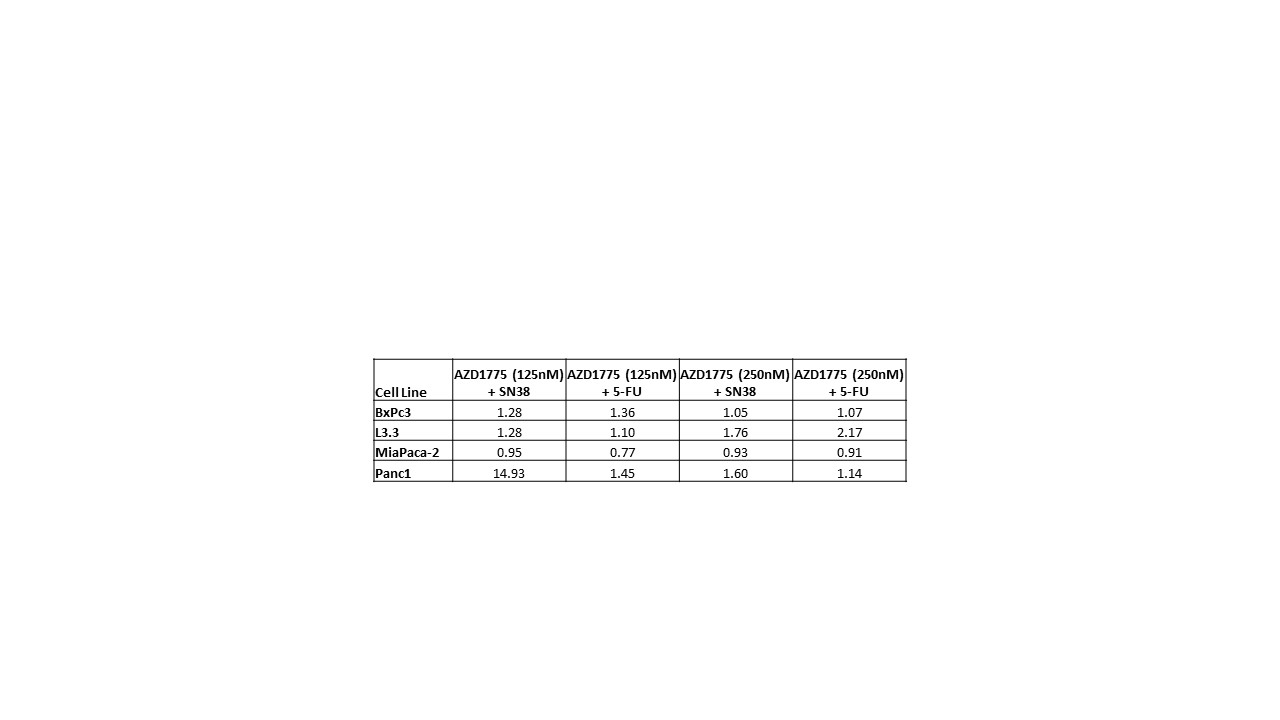

Supplement: Supplementary Table 1 — The synergistic effects of AZD1775 (125 nM, 250 nM) and SN38 or 5-FU in PDAC cell lines. Bliss additivity was calculated using the 72 hour proliferation data averages. Values greater than 1 indicate synergy between the two agents tested. [file Image_4.jpeg]
